# Chemometric tools for the authentication of cod liver oil based on nuclear magnetic resonance and infrared spectroscopy data

**DOI:** 10.1007/s00216-019-02063-y

**Published:** 2019-08-10

**Authors:** Editha Giese, Sascha Rohn, Jan Fritsche

**Affiliations:** 1 Federal Research Institute of Nutrition and Food, Department of Safety and Quality of Milk and Fish Products, Max Rubner-Institut, Hermann-Weigmann-Strasse 1, 24103 Kiel, Germany; 2 Hamburg School of Food Science, Institute of Food Chemistry, University of Hamburg, Grindelallee 117, 20146 Hamburg, Germany; 3 Faculty of Life Sciences/Food Science, Hamburg University of Applied Sciences, Ulmenliet 20, 21033 Hamburg, Germany

**Keywords:** Nuclear magnetic resonance spectroscopy, Infrared spectroscopy, Artificial neural networks, Authenticity, Fish oil, Adulteration

## Abstract

Cod liver oil is a popular dietary supplement marketed as a rich source of omega-3 fatty acids as well as vitamins A and D. Due to its high market price, cod liver oil is vulnerable to adulteration with lower priced vegetable oils. In this study, ^1^H and ^13^C nuclear magnetic resonance spectroscopy, Fourier transform infrared spectroscopy, and gas chromatography (coupled to a flame ionization detector) were used in combination with multivariate statistics to determine cod liver oil adulteration with common vegetable oils (sunflower and canola oils). Artificial neural networks (ANN) were able to differentiate adulteration levels based on infrared spectra with a detection limit of 0.22% and a root mean square error of prediction (RMSEP) of 0.86%. ANN models using ^1^H NMR and ^13^C NMR data yielded detection limits of 3.0% and 1.8% and RMSEPs of 2.7% and 1.1%, respectively. In comparison, the ANN model based on fatty acid profiles determined by gas chromatography achieved a detection limit of 0.81% and an RMSEP of 1.1%. The approach of using spectroscopic techniques in combination with multivariate statistics can be regarded as a promising tool for the authentication of cod liver oil and may pave the way for a holistic quality assessment of fish oils.

**Electronic supplementary material:**

The online version of this article (10.1007/s00216-019-02063-y) contains supplementary material, which is available to authorized users.

**Figure:**
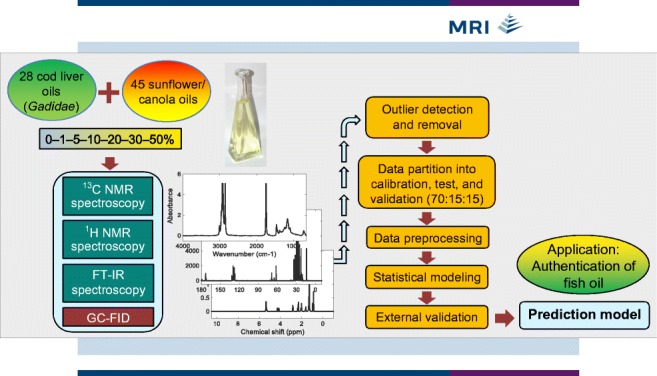
Graphical abstract

## Introduction

Food authentication is the verification that a food complies with its labeled information such as origin, production method, and composition. The labeling of high-value products is of particular interest, as such products may become targets of fraudulent activities [1]. Food fraud has been practiced in almost every period of human history. However, in recent years, it has become more visible because of an increase of global commodity trade. The European Union (EU) regulation no. 882/2004, which lays down rules for the performance of official food controls within the EU, has recently been replaced by regulation 2017/625 [2]. This regulation introduced “fraudulent or deceptive practices” as a new key element in official controls that must be taken into account by the competent authorities and integrated into the risk-based approach for the determination of the frequency of controls.

Although the majority of food fraud incidents do not pose a health risk to the consumer, some cases can cause serious harm to human health, e.g., when expensive fish species are substituted by species associated with certain types of food poisoning or allergens [3]. However, even when it does not entail any health implications, food fraud undermines consumer confidence and may affect an entire food industry sector. As a result, the development of reliable, rapid, and cost-effective analytical authentication methods is currently an area of significant interest for the food industry as well as the food retail sector and food authorities [3].

Traditionally, methods for fish oil authentication are based on chromatography, mass spectrometry, or wet-chemical procedures [4–6], which are often laborious and time-consuming. The *Codex Standard for Fish Oils* defines fish species–specific ranges for individual fatty acids determined by gas chromatography (GC) [7]. *The European Pharmacopoeia* lays down the regiospecific distribution of DHA, EPA, and stearidonic acid on the glycerol backbone of triacylglycerols for the authentication of salmon oil [8]. Fatty acid and triacylglycerol profiles have been successfully applied in combination with *principal component analysis* in order to distinguish between fish and marine mammal oils [4] as well as authentic and adulterated cod liver oils [6]. On the other hand, spectroscopic techniques, such as ^1^H nuclear magnetic resonance (NMR), ^13^C NMR, and Fourier transform infrared (FT-IR) spectroscopies, are known as fingerprint methods that are suitable for non-targeted analyses, especially when they are used in combination with chemometrics. They are considered rapid and non-destructive methods that can provide comprehensive information in a single analysis. Various compositional edible oil quality parameters (e.g., acidity, iodine, peroxide, and saponification values, trans fatty acid content, fatty acid composition) can be determined from the spectra, which makes spectroscopic fingerprint methods suitable for the development of a holistic quality assessment approach [9, 10].

However, the vast amount of data acquired by spectroscopic instruments is complex and difficult to interpret. Traditional methods of calibration based on univariate analysis may not yield satisfactory results. In this case, chemometrics can help to extract the relevant information using mathematical and statistical methods, e.g., machine learning [9]. Machine learning is the scientific discipline of the development and application of algorithms designed to “learn” from training data to create models that can be used to make predictions and/or decisions. Supervised learning comprises classification and regression analyses for qualitative and quantitative approaches, respectively. Linear methods such as *partial least squares regression* (PLSR), *principal component regression* (PCR), and *linear discriminant analysis* (LDA) are most commonly used in food applications [9]. Alternatively, more advanced techniques based on complex algorithms have increasingly attracted attention. These include, e.g., *artificial neural networks* (ANN), *support vector machines*, and *genetic algorithms* [9]. PLSR is often combined with variable selection algorithms, which can be used to select the important variables from the data prior to statistical modeling, such as *Monte Carlo uninformative variable elimination* (MC-UVE) [11], *random frog* [11], and *competitive adaptive reweighted sampling* (CARS) [12].

With regard to food fraud incidences, cod liver oil seems to be an attractive target due to its high nutritional (e.g., long-chain omega-3 fatty acids docosahexaenoic acid (DHA) and eicosapentaenoic acid (EPA) as well as vitamins A and D) and economic values [5, 13]. Potential adulterants include vegetable oils, in particular those that cannot easily be distinguished visually from cod liver oil and are less expensive, e.g., sunflower and canola oil [14].

The literature contains a number of studies where NMR or FT-IR spectroscopy was applied in combination with multivariate statistics to detect olive oil adulteration [15, 16]. ^1^H and ^13^C NMR spectroscopies have been used to classify fish (oil) samples according to species, geographical origin, or farmed and wild status [17, 18]. Two studies using ^13^C NMR spectroscopy investigated the adulteration of salmon oil with mixed fish oil [17] and the adulteration of vegetable oils with animal oils [19]. Only Aursand et al. [17] applied supervised learning to build a regression model for the prediction of adulteration levels in the range 5–40%. A series of studies on the determination of cod liver oil adulteration based on FT-IR spectra were published by the same research group [14, 20–22]. They used various animal fats and vegetable oils as adulterants and applied the linear methods PLSR, PCR, LDA, and *partial least squares discriminant analysis* for modeling. The adulteration levels with vegetable oils analyzed in those studies ranged from 1 to 50% (v/v) [14, 20].

The present study investigated the following hypothesis: The adulteration of cod liver oil with common vegetable oils (here: sunflower and canola oils) can be detected at low levels (< 5%) and quantified by means of ^1^H NMR, ^13^C NMR, and FT-IR spectroscopies in combination with multivariate statistics. Confirming this hypothesis would suggest a potential of the spectroscopic techniques to be used as alternative tools to GC-based methods, which would be beneficial with regard to analysis time, workload, the amounts of sample and chemicals required, and the amount of additional information obtained in the same analysis.

The specific objectives of the study were (a) to generate regression and classification models using a variety of learning algorithms for the determination of cod liver oil adulteration based on the spectroscopic data, (b) to compare the performance of the various linear and non-linear models in order to identify the most suitable statistical methods and the most powerful spectroscopic technique for the issue investigated, and (c) to evaluate the novel spectroscopic approaches against models based on the fatty acid profiles determined by the standard method GC-FID.

## Materials and methods

### Oil samples

A total of 28 different cod liver oils (marine fish family *Gadidae*) were used in this study, of which 13 oils were crude, ten were refined, and five had been filtrated by activated carbon to remove dioxins (Table 1). Samples were provided by Lipromar GmbH (Cuxhaven, Germany) and LYSI hf. (Reykjavik, Iceland). For producing model blends, 27 commercial sunflower oils were used, of which 24 were refined, two were cold-pressed, and one was crude. Seventeen canola oils were used, including 14 refined and three cold-pressed oils. Two of the sunflower oils and one of the canola oils were high-oleic oils. Additionally, one refined, commercially available mixed oil consisting of sunflower and canola was used.

**Table 1:** Cod liver oils analyzed in this study

Raw material	Specification
Frozen liver from *Gadus morhua*	North-East Atlantic, processed in 2013, crude
Frozen liver from *Gadus morhua*	North-East Atlantic, processed on 30 April 2014, crude
Frozen liver from *Gadus morhua*	North-East Atlantic, processed on 01 May 2014, crude
Fresh liver from *Gadus morhua*	Barents Sea/Norwegian Sea, processed on 13 May 2014, crude
Fresh liver from *Gadus morhua*	Norwegian Sea/Bering Sea, North-East Atlantic, processed on 05 March 2015, crude
Fresh liver from *Gadus morhua* (2 different batches)	Barents Sea, processed on 10 March 2015, filtrated by activated carbon
Fresh liver from *Gadus morhua*	Barents Sea/Norwegian Sea, processed on 30 March 2015, crude
Fresh liver from *Gadus morhua*	Barents Sea/Norwegian Sea, processed on 23 April 2015, crude
Fresh liver from *Gadus morhua*	Barents Sea/Norwegian Sea, processed on 23 April 2015, filtrated by activated carbon
Fresh liver from *Gadus morhua*	Barents Sea/Norwegian Sea, processed on 21 May 2015, filtrated by activated carbon
Fresh liver from *Gadus morhua*	Barents Sea/Norwegian Sea, processed on 18 January 2016, crude
Fresh liver from *Gadus morhua*	Processed on 24 March 2016, crude
Fresh liver from *Gadus morhua*	Processed on 02 April 2016, filtrated by activated carbon
Frozen liver from *Gadus chalcogrammus*	North-East Pacific, processed on 08 July 2016, crude
Fresh liver and flesh from *Gadus morhua, Melanogrammus aeglefinus,* and *Pollachius virens* (2 different batches)	Iceland, processed in 2017, refined
Fresh liver from *Gadus morhua*, *Melanogrammus aeglefinus*, and *Pollachius virens* (2 different batches)	Iceland, processed in 2017, refined
Fresh liver from *Gadus morhua*	Greenland Sea, North-West Atlantic, processed on 07 June 2017, crude
Frozen liver from *Gadus morhua*, salted	Greenland Sea, North-West Atlantic, processed on 15 August 2017, crude, supernatant
Frozen liver from *Gadus morhua*, salted	Greenland Sea, North-West Atlantic, processed on 15 August 2017, crude
Fresh liver and flesh from *Gadus morhua*, *Melanogrammus aeglefinus*, and *Pollachius virens* (3 different batches)	Iceland, processed on 30 October 2017, refined
Fresh liver from *Gadus morhua*, *Melanogrammus aeglefinus*, and *Pollachius virens* (3 different batches)	Iceland, processed on 30 October 2017, refined

Binary blends of sunflower/canola oil in cod liver oil were prepared at concentrations of 1%, 5%, 10%, 20%, 30%, and 50% (v/v) by adding the respective volume of vegetable oil to the cod liver oil and mixing the blend for 1 min using a vortex, for 30 min using a laboratory shaker, and again for 1 min using a vortex.

The pure cod liver oils, the blends, and two samples of pure canola and sunflower oils (both refined) were analyzed by ^1^H NMR, ^13^C NMR, and FT-IR spectroscopies as well as by GC-FID.

### Chemicals

Chloroform-d1 (99.8%, 0.03% TMS) was obtained from Deutero GmbH (Kastellaun, Germany). FAME Mix C4-C24, Supelco PUFA-1 Marine Source, *cis*-6,9,12,15-octadecatetraenoic acid methyl ester (≥ 97.0%), and *cis*-7,10,13,16,19-docosapentaenoic acid methyl ester (≥ 98.0%) from Merck KGaA (Darmstadt, Germany) as well as *cis*-8,11,14,17-eicosatetraenoic acid methyl ester (98%) from Larodan AB (Solna, Sweden) were used as external standards for GC analysis.

### NMR spectroscopy

A total of 300 ± 2 mg oil was weighed into a 2-mL reaction tube. After addition of 700 μL deuterated chloroform, the tube was closed and vortexed, and 600 μL of this solution was placed into a 5-mm-diameter NMR tube (Wilmad-LabGlass, Vineland, USA). The tube was capped and analyzed by NMR spectroscopy (Avance III HD 400 MHz, 5 mm BBI Probe, Bruker BioSpin GmbH, Rheinstetten, Germany) in a one-dimensional ^1^H NMR and a one-dimensional ^13^C NMR experiment with ^1^H decoupling. Both experiments were carried out at 300 K. The following experimental conditions were applied in the ^1^H NMR experiment: spectral width 8223.7 Hz, relaxation delay 4 s, number of scans 16, acquisition time 3.9846 s, pulse width 90°, pulse sequence zg, zero filling 64 k. The acquisition parameters of the ^13^C NMR experiment were as follows: spectral width 24,038.5 Hz, relaxation delay 2 s, number of scans 512, acquisition time 1.3631 s, pulse width 90°, pulse sequence zgpg, zero filling 64 k.

The spectra were acquired using TopSpin 3.2 (Bruker BioSpin GmbH, Rheinstetten, Germany), which performed an automated phase correction. The first preprocessing of the spectra was carried out in MestReNova 10.0 (Mestrelab Research S.L., Santiago de Compostela, Spain). A baseline correction (Bernstein polynomial fit, polynomial order 3) was applied, and the spectra were binned (average sum) at an interval of 0.002 ppm (^1^H NMR spectra) or 0.02 ppm (^13^C NMR spectra). The signals of chloroform (7.234–7.328 ppm) and TMS (− 0.049–0.054 ppm) including their ^13^C satellites (7.004–7.028 ppm and 7.526–7.552 ppm, − 0.154–− 0.144 ppm and 0.142–0.152 ppm) were cut out of the ^1^H NMR spectra, and the signals of deuterated chloroform (76.673–77.563 ppm) were eliminated from the ^13^C NMR spectra. Finally, the ^1^H NMR spectra consisted of 5617 and the ^13^C NMR spectra of 9455 data points.

Signal allocation was performed using information from scientific literature as well as the database SDBSWeb (http://sdbs.db.aist.go.jp, National Institute of Advanced Industrial Science and Technology, Tokyo, Japan).

### FT-IR spectroscopy

An Agilent 5500t FT-IR spectrometer (Agilent Technologies Inc., Santa Clara, USA) equipped with a detector of deuterated triglycine sulfate was used to obtain FT-IR spectra. An oil droplet was placed on a zinc selenide window, and absorbance spectra were recorded in transmittance mode over a path length of 100 μm. The samples were scanned at room temperature at a resolution of 4 cm^−1^ in the wavenumber range 4001–649 cm^−1^, whereby 898 data points were generated. The spectra were averaged over 32 scans and ratioed against an air background spectrum which was also averaged over 32 scans. A new background spectrum was obtained after each measurement. The zinc selenide window was cleaned with toluol before a new sample was applied. OPUS 7.2 (Bruker Optik GmbH, Ettlingen, Germany) was used to acquire data and to perform a rubberband baseline correction with 100 baseline points. Average spectra of duplicate measurements were used for statistical analysis.

### Gas chromatographic analysis

Fatty acid methyl ester (FAME) composition (weight%) was determined on a 6890N gas chromatograph (Agilent Technologies Inc., Santa Clara, USA) equipped with an SP-2560 column (100 m × 0.25 mm internal diameter, 0.20 μm film) (Supelco Inc., Bellefonte, USA) and a flame ionization detector (FID) according to the standard method C-VI 10a (00) of the *German Society for Fat Science* (DGF) after alkaline transesterification to FAME (C-VI 11d (98)) [23]. Gas flows were as follows: carrier gas (H_2_) 1.4 mL/min, make-up gas (N_2_) 45 mL/min, detector flame 40 mL/min for H_2_ and 390 mL/min for synthetic air. The injected sample volume was 1.0 μL at a 75:1 split ratio. Injector and detector temperatures were 250 °C. The chromatographic analysis time was 55 min. The oven temperature program comprised heating at 3 °C/min from 120 to 240 °C and holding 240 °C for 15 min. FAME peaks were identified by comparison of their retention times with the retention times of external analytical standards. ChemStation Rev. B 03.02 (Agilent Technologies Deutschland GmbH, Waldbronn, Germany) was used to determine retention times and peak areas. Thirty-seven fatty acids were identified in the analyzed samples. The average values of duplicate measurements were used for statistical analysis.

### Statistical analysis

Various chemometric methods were used to generate models that can determine the adulteration of cod liver oil based on ^1^H NMR, ^13^C NMR, and FT-IR spectra as well as fatty acid profiles determined by GC-FID. The data acquired by the three spectroscopic techniques and the FAME composition (weight%) calculated from the GC chromatograms served as predictor variables, respectively, while the adulteration level (vol.%) and the adulteration status (yes/no) were used as the dependent variable for regression and classification models, respectively.

#### Data preprocessing

Data preprocessing was carried out in MATLAB 9.0 (The MathWorks^®^, Natick, USA). The data was transformed by logarithmization, mean centering, autoscaling, standard normal variate correction, first and second derivative, smoothing, multiplicative scatter correction, min–max normalization from 0 to 1, or combinations thereof. Outliers were detected by the *Monte Carlo sampling method* [11] and removed from the data sets. Two samples (the pure canola and sunflower oils) were excluded as outliers. Subsequently, the data set was divided into a calibration (70%), a test (15%), and a validation (15%) set, which corresponds to the default data partition for using ANN in MATLAB. Calibration samples were used to train the models, while test samples served to optimize model parameters, and validation samples were used for an external validation of the final optimized models. Samples were only eligible for the test and validation sets if the contained cod liver oils were not present in any of the calibration samples. Additionally, samples in the validation set did not contain any cod liver oils that had been used to produce the blends present in the test set. Furthermore, the validation data set had to comprise samples from six concentration categories (0%, 1%, 5%, 10%, 20%, 30%). An exception was the validation set for the GC model, which did not contain any samples with 10% adulteration.

In most data mining applications, only a small fraction of the large number of independent variables that have been measured are indeed relevant for prediction [24]. This is particularly true for spectral data. Variable selection prior to statistical modeling may improve model performance and reduce the computation time for modeling. Therefore, various statistical methods were applied in order to select the most important variables from the data. These methods included *successive projections algorithm* (SPA) [25], MC-UVE (elimination of all variables with stability ≤ 1), *random frog* (initially two variables to sample, variable assessment by regression coefficient), CARS, and *stepwise multiple linear regression* [26]. Moreover, the attribute selection filter in Weka 3.8.2 (University of Waikato, Hamilton, New Zealand) was applied. These methods used the adulteration level (vol.%) as the dependent variable. PLSR (SIMPLS algorithm, mean centering of dependent variable, 50 factors) was implemented in MATLAB as an alternative to the variable selection methods or as an additional tool applied after variable selection in order to reduce the dimensionality of the data.

#### Regression models

The original as well as the preprocessed data served as predictors for various learning algorithms in Weka:

- *Support vector regression*
- *Linear regression*
- *Multivariate adaptive regression splines* (MARS)
- Two instance-based algorithms: *K-nearest neighbors* and *locally weighted regression*
- Three decision tree–based algorithms: *M5′ model tree*, *random forest*, and *REPTree*
- Two multilayer feed-forward ANN: *Bayesian regularized neural network* and *multilayer perceptron*
- Three ensemble methods: *bagging*, *stacking*, and *voting*

The methods MARS and *Bayesian regularized neural network* were implemented in R 3.5.0 (The R Foundation for Statistical Computing, Vienna, Austria) and accessed via Weka.

MARS is a technique which can model non-linearities by building a piecewise linear model [24]. It partitions the range of predictor values into a number of bins and creates a linear regression model for each region. The individual regression models are connected by knots so that the resulting model is continuous. MARS is suitable for high-dimensional problems with a large number of inputs and works very fast. On the other hand, it may not be as accurate as more advanced non-linear algorithms [24].

ANN are a set of mathematical methods mimicking the functioning of the human brain [24]. They consist of artificial neurons arranged in interconnected layers, an input and an output layer, and, where appropriate, one or more hidden layers. Depending on the type of network, they can be used for regression or classification purposes. The major advantage of ANN is that they are capable of both linear and non-linear modeling and can be flexibly adapted to a specific problem in terms of architecture, learning algorithm, activation functions, cost function, etc. However, in cases where spectral data is used as input, variable selection may be required prior to modeling in order to reduce the time to train the network. Furthermore, validation of the models using an external validation set is vital because ANN are prone to overfitting as the ratio of training samples to the number of connection weights is often considered too small. Another drawback is that ANN models may be difficult to interpret, in particular when they consist of many hidden neurons or even several hidden layers [24].

The prediction models generated using different forms of data preprocessing were optimized based on the *root mean square error* (RMSE) obtained for the calibration and test data sets. The optimized models were validated on the validation data set. Eventually, the RMSE (in particular the *root mean square error of prediction* (RMSEP) of the validation data), the bias, the predictive coefficient of determination (*Q*^2^) of the validation data, the limit of detection (LOD), and the limit of quantification (LOQ) were used to evaluate model performance. The LOD and the LOQ were determined in accordance with the *AOAC Guidelines for Single-Laboratory Validation of Chemical Methods for Dietary Supplements and Botanicals* [27]. The LOD was defined as the blank value plus three times the standard deviation of the blank, and the LOQ as the blank value plus ten times its standard deviation. The blank value was defined as the average of at least four model predictions of the adulteration level of pure cod liver oil samples.

#### Classification models

Prediction models were also built on the preprocessed and the original data after conversion of the dependent variable to binary level (adulteration—yes/no). The following classifiers were used in Weka:

- *Naive Bayes*
- *Support vector classification*
- *Logistic regression*
- Two instance-based classifiers: *K-nearest neighbors* and *locally weighted learning*
- Two rule-based classifiers: *repeated incremental pruning to produce error reduction* (RIPPER) and PART, which obtains rules from partial decision trees
- Three decision tree–based classifiers: *C4.5*, *random forest*, and *REPTree*
- Two multilayer feed-forward ANN: *multilayer perceptron* and *voted perceptron*
- Two ensemble methods: *Adaboost.M1* and *bagging*

*Support vector machines* try to find the hyperplane that produces the largest margin between the training points of two classes [24]. The position of this hyperplane is defined by the support vectors, i.e., the data points that are closest to the hyperplane. Using the *kernel trick*, they are also capable of drawing non-linear boundaries between classes by mapping the data into a higher-dimensional feature space where a linear separation becomes possible. However, they are highly affected by outliers and difficult to interpret [24].

Rule learners are fast and usually generate sparse models because they select only the most important variables. Furthermore, decision rules are robust against outliers [28].

In addition to the aforementioned methods, *flexible discriminant analysis* (FDA) using the MARS algorithm and two multilayer feed-forward ANN (neuralnet and nnet) were implemented in R and accessed via Weka. While LDA separates two classes by a hyperplane, FDA uses MARS functions to define the discriminant surface [24].

All of the abovementioned methods were also tested after the application of a filter in Weka that discretizes a range of numeric independent variables into nominal attributes using a supervised discretization method (Fayyad & Irani’s MDL method). The classification models generated using different forms of data preprocessing were optimized based on the percentage of correct classification of the calibration and test set samples with particular emphasis on samples with low adulteration levels.

## Results and discussion

Rohman and Che Man presented three approaches for the detection of adulterants in edible fats and oils [21]: (1) determining the ratios between chemical constituents, while assuming that these ratios are constant for authentic fats and oils; (2) identifying specific lipid markers (e.g., steroids, secondary plant metabolites), which may be present in different concentrations in the pure oils; and (3) applying analytical methods derived from physical analysis to determine the effects of adulteration on the physicochemical properties of the fats and oils. The present study applied the third approach; i.e., it derived information on a potential adulteration from spectroscopic data by building regression (“Regression models”) and classification (“Classification models”) models. The Weka files containing the individual model configurations can be obtained from the authors along with specifications of the PLS factors. These files allow anyone to apply these models to unknown samples of cod liver oil in order to decide whether and/or to which extent they are adulterated.

### Regression models

**Fig. 1:**
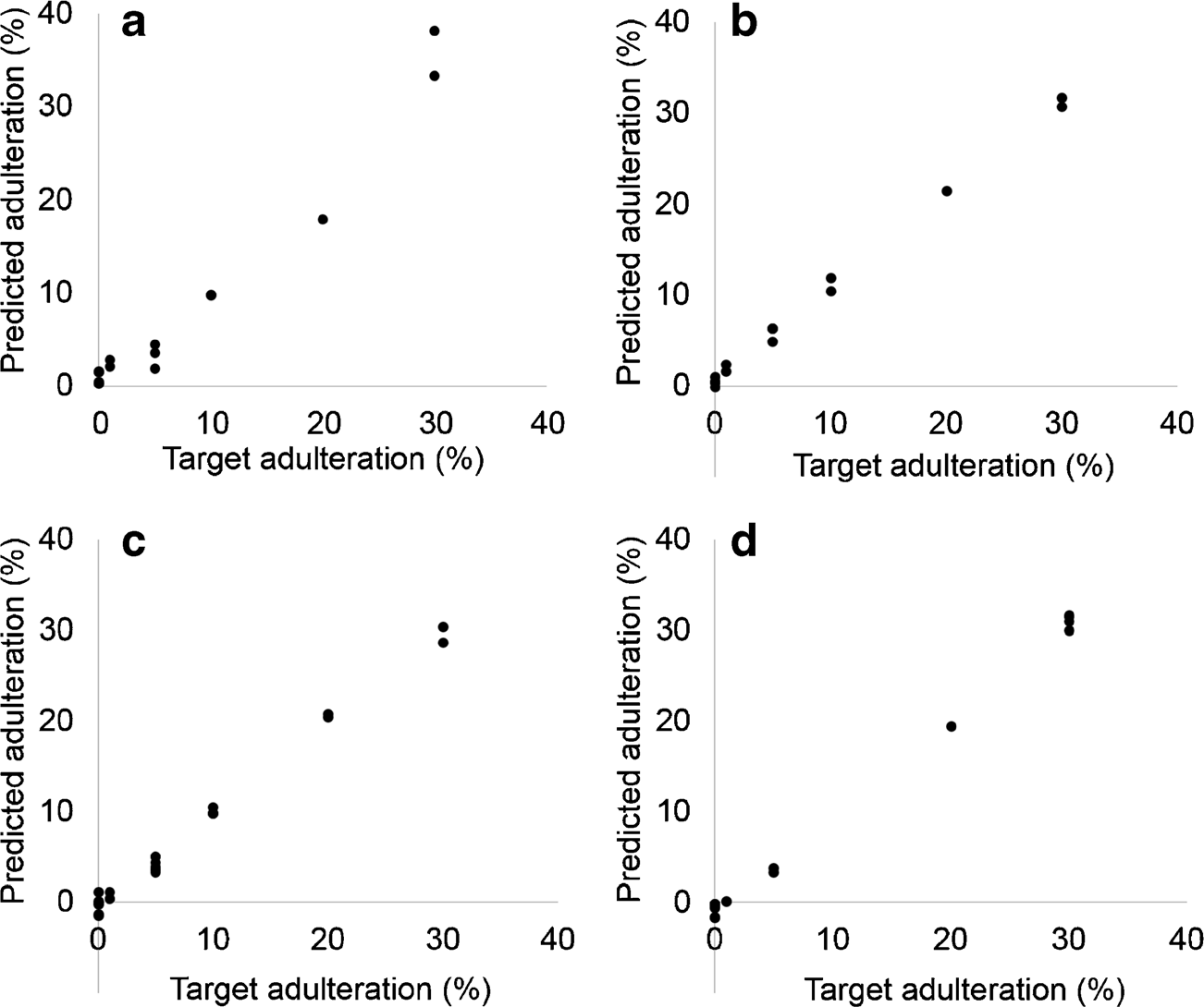
Adulteration level predicted in the validation by the best regression models versus target adulteration: **a**^1^H NMR model; **b**^13^C NMR model; **c** FT-IR model; **d** GC-FID model

The best regression models built on the four data types are presented in Table 2. In all cases, ANN models displayed the best performance. The model based on FT-IR spectra yielded the lowest RMSEP in the validation and the lowest LOD (0.86% and 0.22%, respectively), followed by the GC-FID model (RMSEP = 1.1%, LOD = 0.81%) (Table 2). Figure 1 shows a plot of the adulteration level predicted in the validation by these models against the target value. The performance of the GC-FID model might be further improved if more (minor) fatty acids (e.g., fatty acid isomers) were identified in the chromatograms and included in the model, such as vaccenic, gadoleic, heneicosapentaenoic, and *cis*-7-octadecenoic acid [4].

**Table 2:** Performance of the best regression models based on ^1^H NMR, ^13^C NMR, FT-IR spectroscopies, and fatty acid profiles determined by GC-FID

	Calibration			Test			Validation					
	*n*	RMSEC (%)	*R*^2^	*n*	RMSEP (%)	*Q*^2^	*n*	RMSEP (%)	*Q*^2^	Bias	LOD (%)	LOQ (%)
^1^H NMR	67	0.46	0.999	14	1.5	0.987	14	2.7	0.930	− 0.77	3.0	7.7
^13^C NMR	67	0.30	1.000	14	0.74	0.993	14	1.1	0.989	− 0.90	1.8	5.0
FT-IR	107	0.46	0.999	23	0.59	0.998	23	0.86	0.991	0.35	0.22	2.5
GC-FID	57	0.52	0.998	18	0.52	0.996	15	1.1	0.993	0.36	0.81	2.9

*n* number of samples

*Q*^2^ predictive coefficient of determination

*R*^2^ coefficient of determination

Table 3 describes the ANN models with regard to data preprocessing as well as ANN architecture and training. PLS factors served as inputs to the ANN. The number of factors was selected based on the lowest RMSE obtained for the calibration and test data sets. The ANN models implemented sigmoid and linear activation functions. While the ^13^C NMR model applied Bayesian regularization in order to improve generalization, the other three models were not regularized. Regularization is a technique that reduces overfitting by preventing the model from having large weights (or coefficients) [24].

**Table 3:** Characterization of ANN regression models (Table 2) based on ^1^H NMR, ^13^C NMR, FT-IR spectroscopies, and fatty acid profiles determined by GC-FID

	Data preprocessing	Inputs to ANN	Network type	Network architecture		Activation function			Learning algorithm	Regularization	No. of epochs
				No. of input neurons	No. of hidden neurons	Input layer	Hidden layer	Output layer			
^1^H NMR	Mean centering – PLSR	9 PLS factors, min–max normalized from 0 to 1	Multilayer perceptron	9	5	Sigmoid	Sigmoid	Linear	Backpropagation (learning rate = 0.3, momentum = 0.2)	–	500
^13^C NMR	Logarithmization, mean centering – *random frog* (maximum 50 factors for cross-validation, 2000 simulations): selection probability ≥ 0.01 (447 variables) – mean centering – PLSR	13 PLS factors, autoscaled	Bayesian regularized neural network	13	2	Sigmoid	Sigmoid	Sigmoid	Backpropagation	Bayesian	1000
FT-IR	Mean centering – *random frog* (maximum 20 factors for cross-validation, 10,000 simulations): selection probability > 0.05 (217 variables) – mean centering – PLSR	13 PLS factors, min–max normalized from 0 to 1	Multilayer perceptron	13	6	Sigmoid	Sigmoid	Linear	Backpropagation (learning rate = 0.3, momentum = 0.2)	–	500
GC-FID	Mean centering – *random frog* (maximum 10 factors for cross-validation, 10,000 simulations): selection probability > 0.1 (32 variables) – mean centering – PLSR	5 PLS factors, min–max normalized from 0 to 1	Multilayer perceptron	5	3	Sigmoid	Sigmoid	Linear	Backpropagation (learning rate = 0.3, momentum = 0.3)	–	500

Decision trees (*M5′ model tree*, *random forest*, *REPTree*) as well as *K-nearest neighbors* produced the worst predictions (data not shown). The application of ensemble learning methods, which generate several individual models and combine the results, did not improve the performance of the individual methods.

Fang et al. [29] established PLSR models to determine adulterations of canola oil with beef tallow or lard using ^1^H NMR spectroscopy and GC-MS. Three different brands of canola oil and animal fats were used in that study. The PLSR models detected adulteration levels of as low as 5% (w/w) at an RMSEP of 0.030% (beef tallow) and 0.016% (lard) for the ^1^H NMR model compared with 0.041% (beef tallow) and 0.038% (lard) for the GC-MS model. Guyader et al. [19] spiked vegetable oils with animal fats/oils comprising butter, fish oils as well as chicken, duck, beef, lamb, pork, and egg fats. They estimated the limit of detection of animal in vegetable oil by ^13^C NMR spectroscopy at around 2% by comparing the spectra of adulterated samples with the variability of genuine spectra. Aursand et al. [17] obtained an RMSE of 1% and 1.8% using ANN and PCR, respectively, to predict the adulteration of salmon oil with mixed fish oil based on ^13^C NMR spectra. The models of their study were trained on three sets of samples prepared at four different adulteration levels (5%, 10%, 20%, and 40%).

Rohman and Che Man [20] studied the adulteration of cod liver oil with canola, corn, soybean, and walnut oils over the range of 1–50% (v/v) using FT-IR spectroscopy in combination with PLSR and PCR. The best RMSEP they obtained for their models (built for each adulterant individually) varied between 1.35% (soybean oil) and 1.75% (canola oil). Rohman et al. [14] analyzed binary mixtures of a (single) cod liver oil with a sunflower, a corn, and a grape seed oil at concentration ranges of 0–50% (v/v) using FT-IR spectroscopy. Their PLSR models yielded an RMSEC of 0.48% and an RMSEP of 0.28% for the quantification of cod liver oil in mixtures with corn oil and an RMSEC of 0.52% and an RMSEP of 0.44% for the analysis of grape seed oil mixtures. The model performance statistics for the sunflower oil mixtures were not reported.

The abovementioned models reported in the literature delivered promising results regarding the spectroscopy-based approach to authenticate edible oils. However, they exhibit certain limitations in that their validation data sets consisted of blends containing the same original oils that were used in the calibration sets or the number of different pure oils and their allocation to calibration and validation sets was not reported.

### Classification models

For the classification approach, three different classifiers turned out to provide the best model for the four data types (Table 4). The best ^1^H NMR and FT-IR models applied *support vector machines* using a normalized polynomial kernel and a linear kernel, respectively. Both models applied regularization via the complexity parameter c. The best ^13^C NMR model was generated by FDA using the MARS procedure*.* Backward pruning served to prevent overfitting. Finally, the best GC-FID model was built using RIPPER, which is a propositional rule learner that applies pruning to avoid overfitting.

**Table 4:** Characterization of the best classification models based on ^1^H NMR, ^13^C NMR, FT-IR spectroscopies, and fatty acid profiles determined by GC-FID

	Data preprocessing	Classifier
^1^H NMR	Min–max normalization from 0 to 1	Support vector classification (sequential minimal optimization, c = 1, normalized quadratic kernel)
^13^C NMR	Logarithmization, mean centering – *random frog* (maximum 50 factors for cross-validation, 2000 simulations): selection probability ≥ 0.01 (447 variables) – mean centering – PLSR 11 factors	FDA using MARS (backward pruning*,* no interaction terms)
FT-IR	Mean centering: *random frog* (maximum 20 factors for cross-validation, 10,000 simulations): selection probability > 0.05 (217 variables) – mean centering – PLSR 11 factors – autoscaling	Support vector classification (sequential minimal optimization, c = 100, linear kernel)
GC-FID	Attribute selection by forward searching using greedy hillclimbing augmented with a backtracking facility and correlation-based feature subset selection (Weka, 7 variables)	RIPPER (pruning, minimum total weight of the instances in a rule = 2)

In comparison, the ^13^C NMR and the FT-IR models yielded the highest accuracy, with 100% correct classification in the calibration, test, and validation set (Table 5). The ^1^H NMR and the GC-FID models misclassified one sample with 0 and 1% adulterant, respectively (Table 5).

**Table 5:** Confusion matrix for the best classification models (Table 4) based on ^1^H NMR, ^13^C NMR, FT-IR spectroscopies, and fatty acid profiles determined by GC-FID

				Predicted class			
		Calibration		Test		Validation	
		Yes	No	Yes	No	Yes	No
		^1^H NMR					
Actual class	Yes	57	0	12	0	10	0
	No	0	10	1	1	0	4
		^13^C NMR					
	Yes	57	0	12	0	10	0
	No	0	10	0	2	0	4
		FT-IR					
	Yes	56	0	16	0	17	0
	No	0	51	0	7	0	6
		GC-FID					
	Yes	29	0	15	0	8	1 [1%*]
	No	0	28	0	3	0	6

Yes: adulterated; no: not adulterated

*Adulteration level of incorrectly classified sample

Naive Bayes, *K-nearest neighbors*, *C4.5*, and PART provided the worst predictions (data not shown), and ensemble learning methods did not perform better than individual algorithms.

### Interpretation of the models

Exemplary ^1^H NMR, ^13^C NMR, and FT-IR spectra of pure cod liver and sunflower oils and their blends can be found in the Electronic Supplementary Material (ESM) (Fig. S1). The clearest differences between cod liver and sunflower oils that can be identified with the naked eye are apparent in the ^13^C NMR spectra in the regions 127–130 ppm and 172–173 ppm, corresponding to the –C=C– and the –C=O region, respectively [30].

The ^1^H NMR signals that are particularly important for the prediction of cod liver oil adulteration can be identified by analyzing the loadings of the PLS factors and their weights in the ANN model. Large positive or negative weights imply a high importance of the respective input variable (PLS factor) for predictions, and large positive or negative factor loadings correspond to a high correlation of the respective spectral region with the PLS factor. Consequently, the first PLS factor in the ^1^H NMR regression model exhibited a strong positive effect on the adulteration predicted by the ANN (data not shown). The signals at 1.24–1.36 ppm, which belong to all but C2 and C3 methylene protons in fatty acids that are not directly adjacent to a methine group, showed highly positive loadings on this factor (ESM Fig. S2a). They are present in all fatty acids apart from DHA and EPA, which do not occur in vegetable oils.

Likewise, the first PLS factor in the ^13^C NMR regression model exhibited a strong positive effect on the adulteration level predicted by the ANN (data not shown). This factor was also among the four factors selected as predictors for the classification model by MARS. The highest positive loading on this factor was found at 29.16 ppm (ESM Fig. S2b) and attributed to methylene resonances of oleic, linoleic, and α-linolenic acid [31]. The highest negative loading was present at 29.28 ppm and assigned to methylene resonances of docosapentaenoic, arachidonic, and gondoic acid [31, 32]. Further positive loadings occurred at 14.04 ppm (terminal methyl group of polyunsaturated omega-6 fatty acids), 62.06 ppm (*sn*-1 and *sn*-3 glycerol carbons on triacylglycerols and *sn*-1 glycerol carbon on 1,2-diacylglycerols), and 68.9 ppm (*sn*-2 glycerol carbon on triacylglycerols) [30, 33]. The resonance of the terminal methyl group of monounsaturated and saturated fatty acids at 14.08 ppm [33] exhibited another negative loading (ESM Fig. S2b).

In the FT-IR regression and classification models, the second PLS factor had a high positive impact on the predictions (data not shown). Two wavenumbers exhibited highly negative loadings on this factor: 1704 cm^−1^ and 3024 cm^−1^ (ESM Fig. S2c). The former is associated with –C=O stretching vibrations in free fatty acids, aldehydes, and ketones [34, 35]. This finding was in agreement with the ^13^C NMR models, which determined a positive effect of the level of triacylglycerols on the predicted adulteration. The signal at 3024 cm^−1^ corresponds to =C–H stretching vibrations of *cis*-double bonds [35]. Differences in this region between cod liver/fish oil and vegetable oils were also identified by Rohman et al. [14], Rohman and Che Man [20], and Yadav and Patel [36].

Rohman et al. [22] constructed a PLSR model for the determination of cod liver oil in binary mixtures with corn oil using the IR region 1480–1375 cm^−1^. They identified this region as being particularly useful for modeling as they found that only cod liver oil contained a band at 1397 cm^−1^. This band is attributed to in-plane bending vibrations of =C–H *cis*-olefinic groups [35] and also exhibited a negative loading on the second PLS factor in the FT-IR-based ANN model generated in the present study. Rohman and Che Man [20] observed differences between cod liver oil and vegetable oils not only at 3007 cm^−1^ but also at 2922 cm^−1^ and 2852 cm^−1^ (–C–H asymmetrical and symmetrical stretching in methylene groups) as well as in the fingerprint region between 1300 cm^−1^ and 1100 cm^−1^ (–C–O stretching and –CH_2_– bending) [20, 34]. In the present study, the region 3020–2820 cm^−1^ was not included in the models as *random frog*, which was applied prior to PLSR, did not select any variables from this range.

Finally, the first PLS factor carried the largest positive weights in the ANN regression model based on the GC-FID data (data not shown). This factor was characterized by highly positive loadings for linoleic and oleic acid and highly negative loadings for gondoic acid, DHA, and palmitoleic acid (ESM Table S1). In the classification approach, the RIPPER algorithm built three rules based on the contents of linoleic and oleic acid. When the linoleic acid content was no higher than 2.005% or the oleic acid content was no higher than 14.205%, the oil was considered to be pure cod liver oil. In all other cases, the oil was classified as a blend. These findings were in agreement with the ^13^C NMR model of the present study, as well as the study of Araujo et al. [4], who applied *principal component analysis* to the fatty acid profiles of different marine and vegetable oils measured by GC-FID. Their results showed that the vegetable oils (soybean, linseed, and canola oils) were characterized by high contents of linoleic and oleic acid, whereas high contents of palmitoleic and gondoic acid were typical for marine oils (cod liver, salmon, seal, and whale oils). However, the absolute values for the upper limits of linoleic and oleic acid contents determined by RIPPER in the present study differed from the ranges specified in the *Codex Standard for Fish Oils*, according to which linoleic acid may be present in cod liver oil at levels of up to 3.0% and oleic acid contents may be as high as 21.0% [7]. This may indicate that the cod liver oils analyzed in this study did not adequately cover the inherent variability.

## Conclusion

This study investigated the hypothesis of whether ^1^H NMR, ^13^C NMR, and FT-IR spectroscopies were suitable to be used in combination with multivariate statistics in order to detect and quantify the adulteration of cod liver oil with vegetable oils (sunflower and canola oils). It was found that *artificial neural networks* were able to determine cod liver oil adulteration based on FT-IR spectra with a detection limit of 0.22% and a *root mean square error of prediction* (RMSEP) of 0.86%. In comparison, the best regression model based on the fatty acid profiles determined by the standard GC method achieved a detection limit of 0.81% and an RMSEP of 1.1%. Moreover, *support vector machines* and *flexible discriminant analysis* using *multivariate adaptive regression splines* were able to achieve 100% correct classification of pure cod liver oils and samples adulterated in the range 1–50% using FT-IR and ^13^C NMR spectra, respectively.

Depending on the desired detection limit and accuracy, the spectroscopic techniques investigated can be regarded as alternative tools to GC-FID in the determination of cod liver oil adulteration, when applied in combination with chemometrics. Moreover, these techniques can be used for a holistic quality assessment of fish oils, as they are able to provide information on various quality aspects and research questions. However, before they can be widely used by fish oil manufacturers and food control authorities, the models should be further validated by incorporating more cod liver oil samples of different geographical origins, seasons, production years, and qualities as well as vegetable oils of more than two botanical origins (including, e.g., soybean and corn oils). This will increase their robustness and their scope of application.

## Electronic supplementary material

ESM 1 (PDF 525 kb)

## References

[1] Danezis GP, Tsagkaris AS, Camin F, Brusic V, Georgiou CA (2016). Food authentication: techniques, trends & emerging approaches. Trends Anal Chem.

[2] European Parliament, Council of the European Union. Regulation (EU) 2017/625 of the European Parliament and of the Council of 15 March 2017 on official controls and other official activities performed to ensure the application of food and feed law, rules on animal health and welfare, plant health and plant protection products, amending Regulations (EC) No 999/2001, (EC) No 396/2005, (EC) No 1069/2009, (EC) No 1107/2009, (EU) No 1151/2012, (EU) No 652/2014, (EU) 2016/429 and (EU) 2016/2031 of the European Parliament and of the Council, Council Regulations (EC) No 1/2005 and (EC) No 1099/2009 and Council Directives 98/58/EC, 1999/74/EC, 2007/43/EC, 2008/119/EC and 2008/120/EC, and repealing Regulations (EC) No 854/2004 and (EC) No 882/2004 of the European Parliament and of the Council, Council Directives 89/608/EEC, 89/662/EEC, 90/425/EEC, 91/496/EEC, 96/23/EC, 96/93/EC and 97/78/EC and Council Decision 92/438/EEC (Official Controls Regulation) (Text with EEA relevance). Off J Eur Union. 2017;L 95:1–142.

[3] Johnson R. Food fraud and “economically motivated adulteration” of food and food ingredients. Congressional Research Service; 2014.

[4] Araujo P, Zeng Y, Du ZY, Nguyen TT, Frøyland L, Grung B (2010). Discrimination of n-3 rich oils by gas chromatography. Lipids..

[5] Tangendjaja B, Davis DA (2015). Quality control of feed ingredients for aquaculture. Feed and feeding practices in aquaculture.

[6] Zeng YX, Araujo P, Du ZY, Nguyen TT, Frøyland L, Grung B (2010). Elucidation of triacylglycerols in cod liver oil by liquid chromatography electrospray tandem ion-trap mass spectrometry. Talanta..

[7] Codex Alimentarius Commission (2017). Standard for fish oils. CODEX STAN 329-2017.

[8] Council of Europe (2005). European Pharmacopoeia.

[9] Ropodi AI, Panagou EZ, Nychas GJE (2016). Data mining derived from food analyses using non-invasive/non-destructive analytical techniques; determination of food authenticity, quality & safety in tandem with Computer Science Disciplines. Trends Food Sci Technol.

[10] Nunes CA (2014). Vibrational spectroscopy and chemometrics to assess authenticity, adulteration and intrinsic quality parameters of edible oils and fats. Food Res Int.

[11] Li HD, Xu QS, Liang YZ (2018). libPLS: an integrated library for partial least squares regression and linear discriminant analysis. Chemom Intell Lab Syst.

[12] Li H, Liang Y, Xu Q, Cao D (2009). Key wavelengths screening using competitive adaptive reweighted sampling method for multivariate calibration. Anal Chim Acta.

[13] Lentjes MAH, Mulligan AA, Welch AA, Bhaniani A, Luben RN, Khaw KT (2015). Contribution of cod liver oil-related nutrients (vitamins A, D, E and eicosapentaenoic acid and docosahexaenoic acid) to daily nutrient intake and their associations with plasma concentrations in the EPIC-Norfolk cohort. J Hum Nutr Diet.

[14] Rohman A, Widyaningtyas R, Amalia F (2017). Authentication of cod liver oil from selected edible oils using FTIR spectrophotometry and chemometrics. Int Food Res J.

[15] García-González DL, Mannina L, D’Imperio M, Segre AL, Aparicio R (2004). Using ^1^H and ^13^C NMR techniques and artificial neural networks to detect the adulteration of olive oil with hazelnut oil. Eur Food Res Technol.

[16] Marigheto NA, Kemsley EK, Defernez M, Wilson RH (1998). A comparison of mid-infrared and Raman spectroscopies for the authentication of edible oils. J Am Oil Chem Soc.

[17] Aursand M, Standal IB, Axelson DE (2007). High-resolution ^13^C nuclear magnetic resonance spectroscopy pattern recognition of fish oil capsules. J Agric Food Chem.

[18] Rezzi S, Giani I, Héberger K, Axelson DE, Moretti VM, Reniero F, Guillou C (2007). Classification of Gilthead sea bream (*Sparus aurata*) from ^1^H NMR lipid profiling combined with principal component and linear discriminant analysis. J Agric Food Chem.

[19] Guyader S, Thomas F, Portaluri V, Jamin E, Akoka S, Silvestre S, Remaud G (2018). Authentication of edible fats and oils by non-targeted ^13^C INEPT NMR spectroscopy. Food Control.

[20] Rohman A, Che Man YB (2011). Application of Fourier transform infrared (FT-IR) spectroscopy combined with chemometrics for authentication of cod-liver oil. Vib Spectrosc.

[21] Rohman A, Che Man YB (2012). Application of Fourier transform infrared spectroscopy for authentication of functional food oils. Appl Spectrosc Rev.

[22] Rohman A, Che Man YB, Ismail A, Puziah H (2011). FTIR spectroscopy combined with multivariate calibration for analysis of cod liver oil in binary mixture with corn oil. Int Food Res J.

[23] DGF (2012). Deutsche Einheitsmethoden zur Untersuchung von Fetten, Fettprodukten, Tensiden und verwandten Stoffen.

[24] Hastie T, Tibshirani R, Friedman J (2009). The elements of statistical learning: data mining, inference, and prediction.

[25] Araújo MCU, Saldanha TCB, Galvão RKH, Yoneyama T, Chame HC, Visani V (2001). The successive projections algorithm for variable selection in spectroscopic multicomponent analysis. Chemom Intell Lab Syst.

[26] Forina M, Lanteri S, Casale M, Cerrato Oliveros MC (2007). Stepwise orthogonalization of predictors in classification and regression techniques: an “old” technique revisited. Chemom Intell Lab Syst.

[27] AOAC Int. Appendix K: guidelines for dietary supplements and botanicals – PART I: AOAC guidelines for single-laboratory validation of chemical methods for dietary supplements and botanicals. In: AOAC Int. AOAC Official Methods of Analysis. AOAC Int; 2013. pp. 1–15.

[28] Molnar C. Decision rules. In: Interpretable machine learning: a guide for making black box models explainable. 2019. https://christophm.github.io/interpretable-ml-book/rules.html. Accessed 15 May 2019.

[29] Fang G, Goh JY, Tay M, Lau HF, Li SF (2013). Characterization of oils and fats by ^1^H NMR and GC/MS fingerprinting: classification, prediction and detection of adulteration. Food Chem.

[30] Siddiqui N, Sim J, Silwood CJL, Toms H, Iles RA, Grootveld M (2003). Multicomponent analysis of encapsulated marine oil supplements using high-resolution ^1^H and ^13^C NMR techniques. J Lipid Res.

[31] Aursand M, Grasdalen H (1992). Interpretation of the ^13^C-NMR spectra of omega-3 fatty acids and lipid extracted from the white muscle of Atlantic salmon (*Salmo salar*). Chem Phys Lipids.

[32] Gunstone FD (1991). High resolution NMR studies of fish oils. Chem Phys Lipids.

[33] Fiori L, Solana M, Tosi P, Manfrini M, Strim C, Guella G (2012). Lipid profiles of oil from trout (*Oncorhynchus mykiss*) heads, spines and viscera: trout by-products as a possible source of omega-3 lipids?. Food Chem.

[34] Guillén MD, Cabo N (1997). Infrared spectroscopy in the study of edible oils and fats. J Sci Food Agric.

[35] Vlachos N., Skopelitis Y., Psaroudaki M., Konstantinidou V., Chatzilazarou A., Tegou E. (2006). Applications of Fourier transform-infrared spectroscopy to edible oils. Analytica Chimica Acta.

[36] Yadav R, Patel PN (2016). Experimental study of adulteration detection in fish oil using novel PDMS cavity bonded EBG inspired patch sensor. IEEE Sensors J.

