# Correction to: Chemometric tools for the authentication of cod liveroil based on nuclear magnetic resonance and infrared spectroscopy data

**DOI:** 10.1007/s00216-019-02122-4

**Published:** 2019-10-19

**Authors:** Editha Giese, Sascha Rohn, Jan Fritsche

**Affiliations:** 1 Federal Research Institute of Nutrition and Food, Department of Safety and Quality of Milk and Fish Products, Max Rubner-Institut, Hermann-Weigmann-Strasse 1, 24103 Kiel, Germany; 2 Hamburg School of Food Science, Institute of Food Chemistry, University of Hamburg, Grindelallee 117, 20146 Hamburg, Germany; 3 Faculty of Life Sciences/Food Science, Hamburg University of Applied Sciences, Ulmenliet 20, 21033 Hamburg, Germany

**Correction to: Analytical and Bioanalytical Chemistry**

10.1007/s00216-019-02063-y

The article Chemometric tools for the authentication of cod liver oil based on nuclear magnetic resonance and infrared spectroscopy data, written by Editha Giese, Sascha Rohn and Jan Fritsche, was originally published electronically on the publisher’s internet portal (currently SpringerLink) on 11 August 2019 without open access.

With the author(s)’ decision to opt for Open Choice the copyright of the article changed on 20 September 2019 to © The Author(s) 2019 and the article is forthwith distributed under the terms of the Creative Commons Attribution 4.0 International License (http://creativecommons.org/licenses/by/4.0/), which permits use, duplication, adaptation, distribution and reproduction in any medium or format, as long as you give appropriate credit to the original author(s) and the source, provide a link to the Creative Commons license and indicate if changes were made.

