# Supplementary material for: Chemometric tools for the authentication of cod liver oil based on nuclear magnetic resonance and infrared spectroscopy data
Source: Anal Bioanal Chem. 2019 Aug 10;411(26):6931–42. doi: 10.1007/s00216-019-02063-y (PMC6834736; doi:10.1007/s00216-019-02063-y)
Supplement: Supplementary file 1 — (PDF 525 kb) [file 216_2019_2063_MOESM1_ESM.pdf]

Analytical and Bioanalytical Chemistry

Electronic Supplementary Material

Chemometric tools for the authentication of cod liver oil based on nuclear magnetic resonance and infrared spectroscopy data

Editha Giese, Sascha Rohn, Jan Fritsche

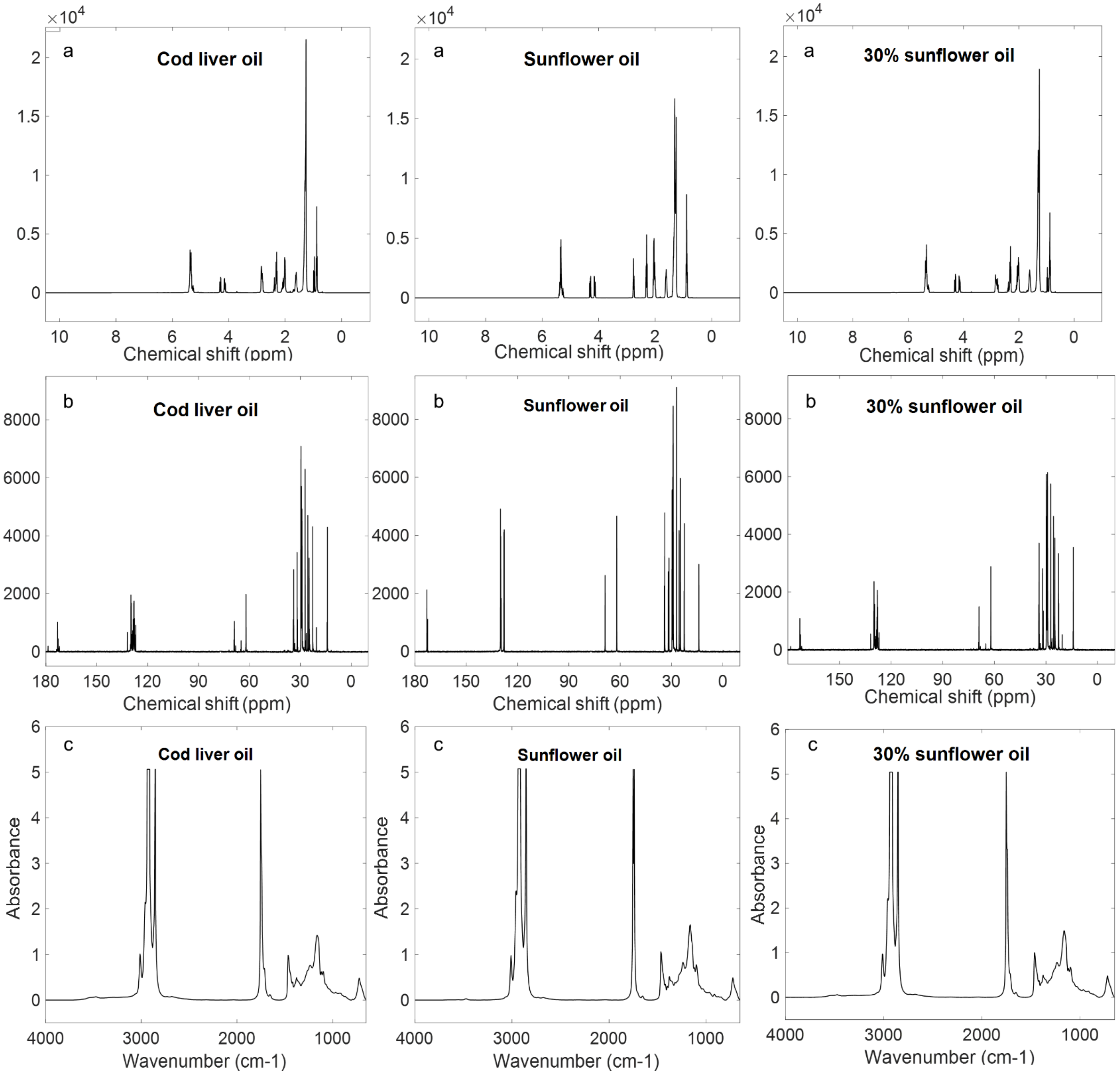

Fig. S1 Exemplary spectra of a crude cod liver oil, a refined sunflower oil, and a blend thereof (30% sunflower oil): a) ^{1}H NMR; b) ^{13}C NMR; c) FT-IR

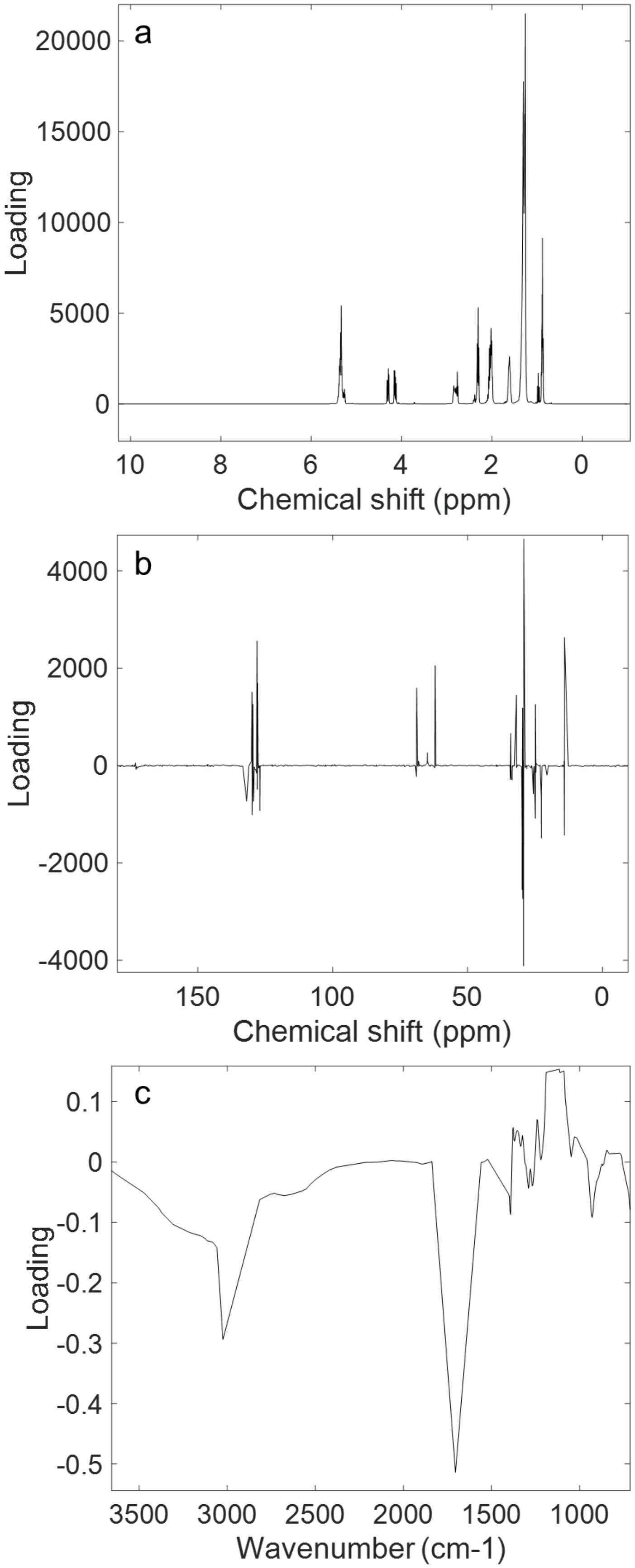

Fig. S2 Factor loadings of PLS factors with high absolute weights in the ANN regression models: a) ^{1}H NMR model (1st PLS factor); b) ^{13}C NMR model (1st PLS factor); c) FT-IR model (2nd PLS factor)

Table S1 Factor loadings of the first PLS factor in the ANN regression model based on fatty acid profiles determined by GC-FID.

Fatty acid	Factor loading	Fatty acid	Factor loading
18:2	43.20	14:1	-0.18
18:1 n-9	24.60	20:3 n-3	-0.19
18:3 alpha	1.10	18:1 t n-9	-0.21
18:0	1.00	17:1	-0.22
22:0	0.50	15:0	-0.28
20:0	0.40	24:1	-0.30
18:3 c,c,t	0.20	20:4 n-6	-0.36
18:2 c,t	0.10	20:2	-0.39
22:2	0.00	20:4 n-3	-0.59
21:0	0.00	22:5 n-3	-0.94
18:3 t,c,t	-0.10	18:4	-2.13
20:3 n-6	-0.10	16:0	-3.33
18:2 t,t	-0.10	14:0	-3.50
18:2 t,c	-0.10	16:1	-8.24
23:0	-0.20	22:6	-10.50
17:0	-0.20	20:1 n-9	-11.00
